# Intracellular Conversion of Environmental Nitrate and Nitrite to Nitric Oxide with Resulting Developmental Toxicity to the Crustacean *Daphnia magna*

**DOI:** 10.1371/journal.pone.0012453

**Published:** 2010-08-27

**Authors:** Bethany R. Hannas, Parikshit C. Das, Hong Li, Gerald A. LeBlanc

**Affiliations:** Department of Environmental & Molecular Toxicology, North Carolina State University, Raleigh, North Carolina, United States of America; East Carolina University, United States of America

## Abstract

**Background:**

Nitrate and nitrite (jointly referred to herein as NO_x_) are ubiquitous environmental contaminants to which aquatic organisms are at particularly high risk of exposure. We tested the hypothesis that NO_x_ undergo intracellular conversion to the potent signaling molecule nitric oxide resulting in the disruption of endocrine-regulated processes.

**Methodology/Principal Findings:**

These experiments were performed with insect cells (*Drosophila* S2) and whole organisms *Daphnia magna*. We first evaluated the ability of cells to convert nitrate (NO_3_^−^) and nitrite (NO_2_^−^) to nitric oxide using amperometric real-time nitric oxide detection. Both NO_3_^−^ and NO_2_^−^ were converted to nitric oxide in a substrate concentration-dependent manner. Further, nitric oxide trapping and fluorescent visualization studies revealed that perinatal daphnids readily convert NO_2_^−^ to nitric oxide. Next, daphnids were continuously exposed to concentrations of the nitric oxide-donor sodium nitroprusside (positive control) and to concentrations of NO_3_^−^ and NO_2_^−^. All three compounds interfered with normal embryo development and reduced daphnid fecundity. Developmental abnormalities were characteristic of those elicited by compounds that interfere with ecdysteroid signaling. However, no compelling evidence was generated to indicate that nitric oxide reduced ecdysteroid titers.

**Conclusions/Significance:**

Results demonstrate that nitrite elicits developmental and reproductive toxicity at environmentally relevant concentrations due likely to its intracellular conversion to nitric oxide.

## Introduction

Nitrogen makes up 78% of the earth's atmosphere and is also the fourth most abundant element in organisms. Atmospheric nitrogen (N_2_, N_2_O) is not biologically available and must be transformed or “fixed” for use in biological processes. Under natural nitrogen cycling conditions, bacterial nitrogen fixation produces nitrate (NO_3_^−^) or ammonium (NH_4_^+^) ions. Nitrifying bacteria oxidize NH_4_^+^ first to nitrite (NO_2_^−^) and then to NO_3_^−^ [1], [2]. Normally, levels of nitrite and nitrate (jointly referred to herein as NO_x_) do not accumulate to excessive levels in the environment because they are assimilated as sources of nitrogen by plants or ultimately converted back to atmospheric nitrogen through bacterial denitrification. As such, biologically available nitrogen is typically a major limiting factor for life on the planet and nitrogen cycling serves as a major regulator of the structure, function, and integrity of ecosystems.

However, human activities have led to drastic increases in the amount of biologically available NO_x_ found in the environment [3], [4]. Anthropogenic sources of NO_x_ pollution include both non-point sources such as runoff from agricultural areas containing manufactured nitrogen fertilizer or nitrate-containing manure generated from concentrated animal feeding operations (CAFOs), and point sources such as municipal wastewater effluents and industrial discharges [1]. NO_x_ are highly water soluble, and thus can readily enter surface and ground waters through rain events. Consequently, aquatic organisms are at high risk for exposure to these compounds.

Human-driven contributions of NO_x_ to the environment are on the rise globally, and therefore contamination of aquatic systems is not only ubiquitous, but also increasing. The United States drinking water standard for nitrate is 10 mg N/L [5]. This drinking water standard is directed largely towards the protection against methemoglobinemia in infants [6]. Relatively little attention has been directed towards potential adverse impacts from chronic, low-level exposure to these compounds by humans or wildlife. Reproduction was significantly reduced during continuous exposure of the water flea (*Ceriodaphnia dubia*) to nitrate concentrations as low as 2 mg N/L [7]. This value stands in contrast to the criterion of 40 mg N/L nitrate established by the Canadian Ministry of Environment for the protection of aquatic life against chronic toxicity [8].

Guillette and Edwards [9] provided compelling evidence that levels of NO_x_ in the environment may be disrupting reproductive physiology of wildlife. They proposed that chronic toxicity of NO_x_ may, in part, be due to: 1) competitive displacement of chloride ions from membrane transporters, 2) binding to the heme groups of steroidogenic enzymes and inhibiting hormone synthesis, or 3) conversion to the potent signaling molecule nitric oxide. In support of the later two proposed mechanisms, Panesar and Chan [10] reported that NO_x_ inhibited steroidogenesis in exposed Leydig cells. These investigators proposed that NO_x_ is converted in the cell to nitric oxide which then binds to the heme group of steroidogenic enzymes and suppresses their catalytic activity. The conversion of environmental NO_x_ to nitric oxide could therefore be a major cause of endocrine disruption in aquatic species where exposure is potentially high. Conversion of NO_x_ to nitric oxide may occur through numerous mechanisms including: non-enzymatic acidic disproportionation [11], mitochondrial cytochrome *c* reductase activity under hypoxic conditions [12], [13], reduction by cytochrome P450s [14], enzymatic conversion by xanthine oxidoreductase [15], [16], reduction by deoxyhemoglobin [17], [18], or conversion by nitrate/nitrite reductase enzymes associated with oral or gastric bacteria [19]. Several of these NO_x_ reduction mechanisms are relevant to aquatic organisms that possess the required biochemical machinery, or live under environmental conditions (hypoxia, low pH) that are conducive to the generation of nitric oxide.

Nitric oxide is a potent, short-lived signaling molecule that regulates a variety of physiological processes. Nitric oxide is normally produced endogenously from L-arginine and molecular oxygen by the enzyme nitric oxide synthase [20]. Nitric oxide can mediate its biological effects by binding to many targets, including heme groups, cysteine residues, and iron and zinc clusters. Since there are numerous targets for nitric oxide-mediated signaling, tight regulation of nitric oxide production is required to maintain normal biological activity. When nitric oxide levels are too high or sustained for too long, as may occur following exposure to nitrates or nitrites, toxicity and disease can occur [21], [22], [23], [24].

We hypothesize that environmental NO_x_ pose risk of chronic toxicity to arthropods through their conversion to nitric oxide. We further hypothesize that nitric oxide interferes with endocrine signaling by lowering ecdysteroid titers. We evaluated the ability of arthropod cells (*Drosophila* S2) and whole organisms (*Daphnia magna*) to convert nitrate and nitrite to nitric oxide. Furthermore, we evaluated whole organism effects of NO_x_ during continuous exposure of *D. magna*. Finally, we evaluated ecdysteroid titers in daphnids following nitric oxide exposure. Arthropods serve as keystone species in many ecosystems and are commonly used as sentinel species for contaminant exposures. Arthropod cells and organisms used in the present study serve as models for evaluating biological interactions with NO_x_ and provide direct information for making decisions regarding the health threat posed to aquatic ecosystems by these compounds.

## Results

### Cellular conversion of NO_x_ to nitric oxide

**Figure 1:**
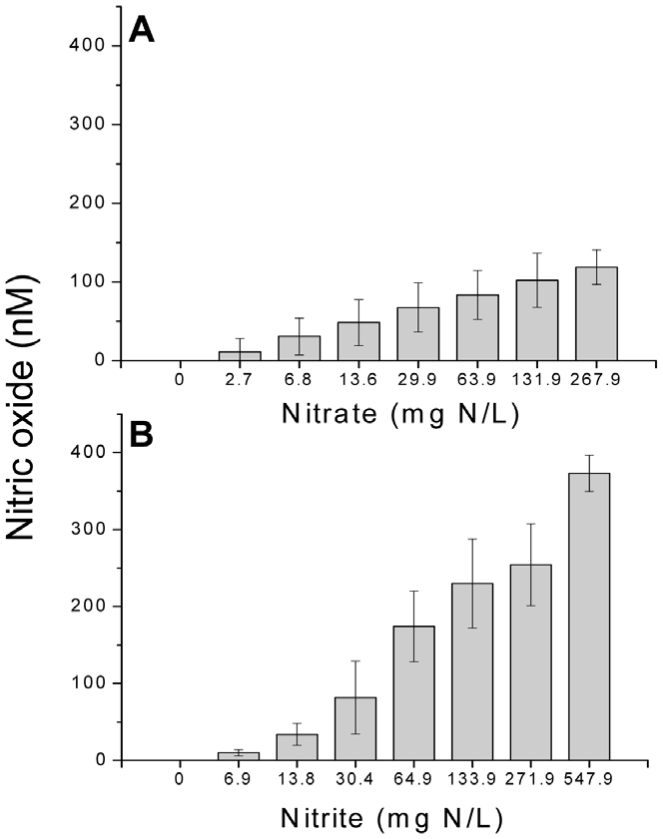
Concentration-dependent conversion of NO_3_^−^ (A) or NO_2_^−^ (B) to nitric oxide by *Drosophila* S2 cells. Each column represents the mean ± SD of 4–7 experiments with different cell batches.

**Figure 2:**
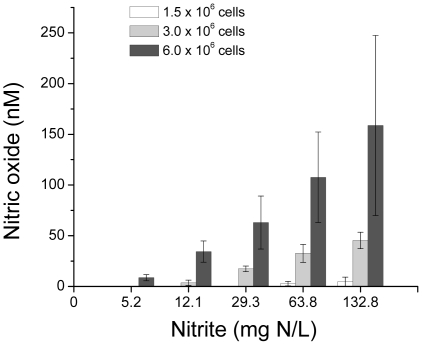
NO_2_^−^-dependent nitric oxide production at increasing cell densities. Each bar represents the mean ± SD of 2–3 experiments with different batches of cells.

*Drosophila* S2 cells were exposed to increasing concentrations of NO_3_^−^ or NO_2_^−^ to determine if these compounds are converted to nitric oxide by arthropod cells. Nitric oxide was generated from NO_x_ in a substrate (Fig. 1) and cell (demonstrated with NO_2_^−^, Fig. 2) concentration-dependent manner. NO_2_^−^ was more efficiently converted to nitric oxide relative to the conversion of NO_3_^−^ due likely to its increased cellular uptake (see Discussion) or requirement for a single reduction reaction as compared to the dual reduction required of NO_3_^−^. Results clearly demonstrate that arthropod cells can convert nitrate and nitrite to nitric oxide.

### Nitric oxide accumulation in perinatal daphnids exposed to NO_x_

**Figure 3:**
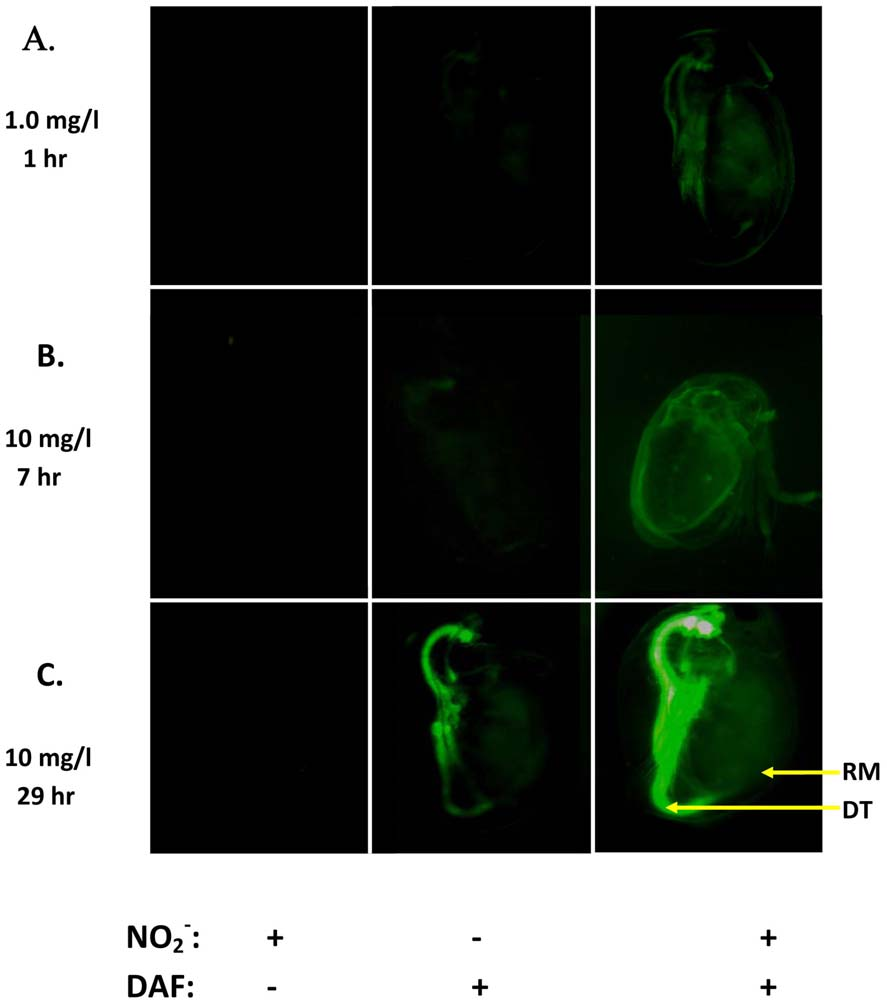
Nitric oxide accumulation in NO_2_^−^-exposed perinatal daphnids as indicated by trapping and fluorescent visualization with diacetylaminofluorocene (DAF, 10 µM). NO_2_^−^ exposure concentration (as mg N/l) and duration of exposure are presented to the left of each row of images. DT and RM denote the digestive tract and respiratory membranes, respectively.

*Ex vivo* exposure of perinatal daphnids to NO_2_^−^ and the nitric oxide-binding fluorescent dye diacetylaminofluorocene (DAF) resulted in appreciably greater internal fluorescence as compared to daphnids exposed to DAF alone (Fig. 3). Fluorescence was largely associated with respiratory membranes and the digestive tract. These are likely the predominant sites of NO_2_^−^ uptake and indicate significant conversion of nitrite to nitric oxide upon entry into the body. Fluorescence associated with the digestive tract appreciably increased in intensity with increasing duration of exposure. This likely reflects the increased passage of NO_2_^−^ and DAF-containing media through the gut with increasing development of the neonates. Unlike NO_2_^−^, NO_3_^−^ failed to increase DAF-associated fluorescence in similarly-performed experiments. We speculate that the assay was insufficiently sensitive to detect the small amount of nitric oxide generated from NO_3_^−^ during the experiments.

### Reproductive and developmental toxicity

**Figure 4:**
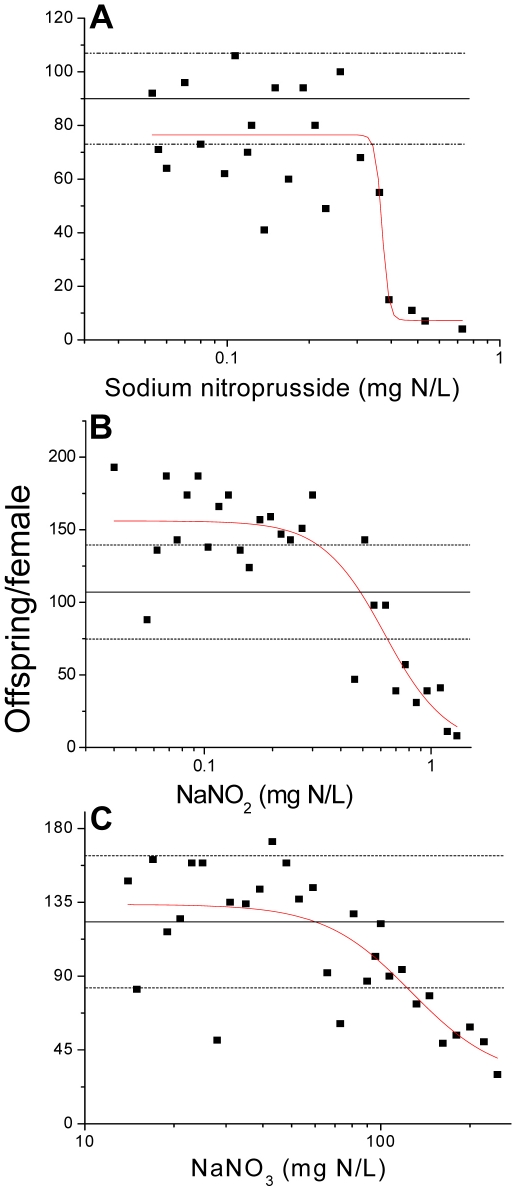
Total offspring released from daphnids chronically exposed to increasing concentration of (A) sodium nitroprusside, (B) NaNO_2_, or (C) NaNO_3_. Each data point represents the total number of offspring released from a single exposed daphnid over the entire exposure period. Mean ± SD negative control performance is depicted by the solid line bracketed by dotted lines.

**Figure 5:**
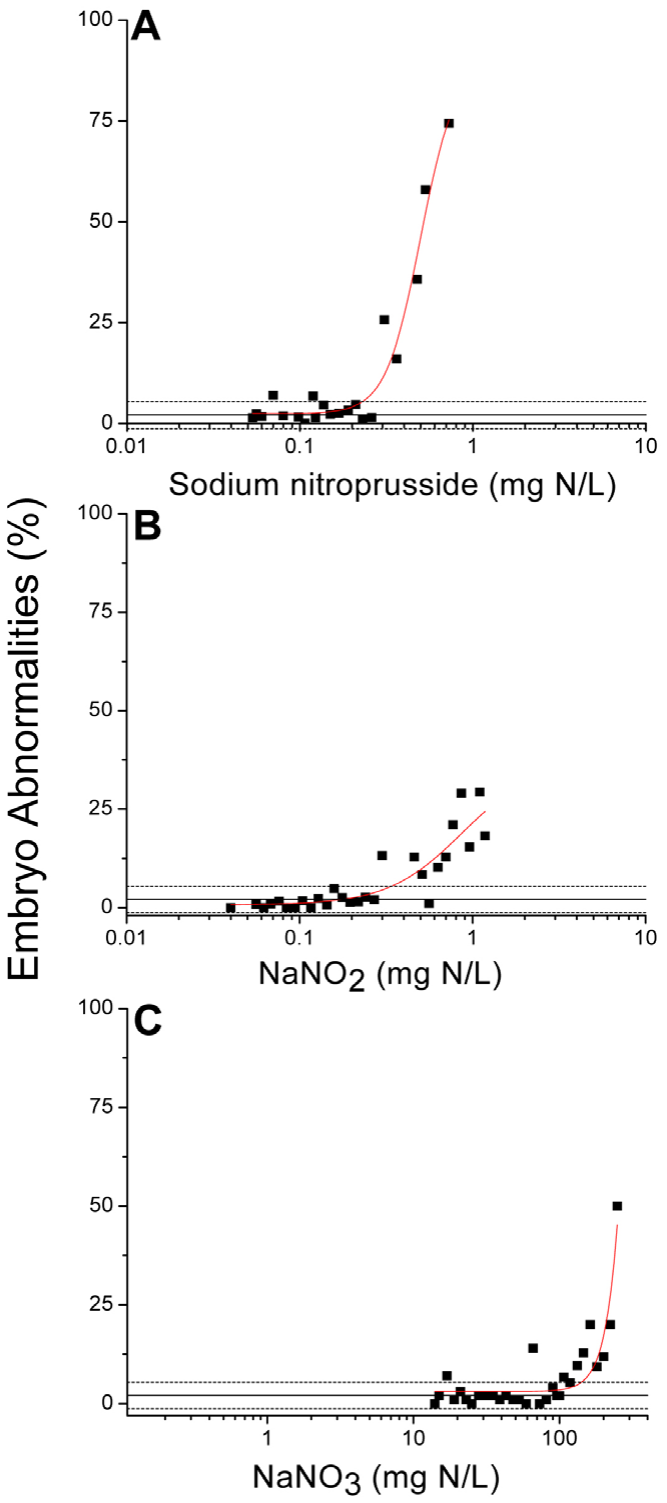
Frequency of neonatal abnormalities among daphnids exposed to increasing concentrations of (A) sodium nitroprusside, (B) NaNO_2_, or (C) NaNO_3_. Each data point represents the percentage of abnormal neonates produced by a single maternal daphnid. Mean ± SD negative control performance is depicted by the solid line bracketed by dotted lines.

Having established that NO_x_ is susceptible to intracellular conversion to nitric oxide, we set out to identify reproductive and developmental toxicity associated with nitric oxide, using sodium nitroprusside as the nitric oxide donor. We also tested whether nitrate and nitrite elicit toxicity that is comparable to that caused by nitric oxide. Sodium nitroprusside reduced the number of offspring produced per maternal daphnid in a concentration-dependent manner with a threshold effect concentration of ∼0.34 mg N/L (Fig. 4A). Sodium nitroprusside also caused the production of abnormally developed offspring (Fig. 5A). Abnormal neonates presented with under-developed second antennae, an un-extended or poorly-extended shell spine, or overall underdeveloped body form. Developmental abnormalities occurred with a threshold concentration of ∼0.23 mg N/L over the same approximate sodium nitroprusside concentration range as observed for reproductive toxicity.

**Figure 6:**
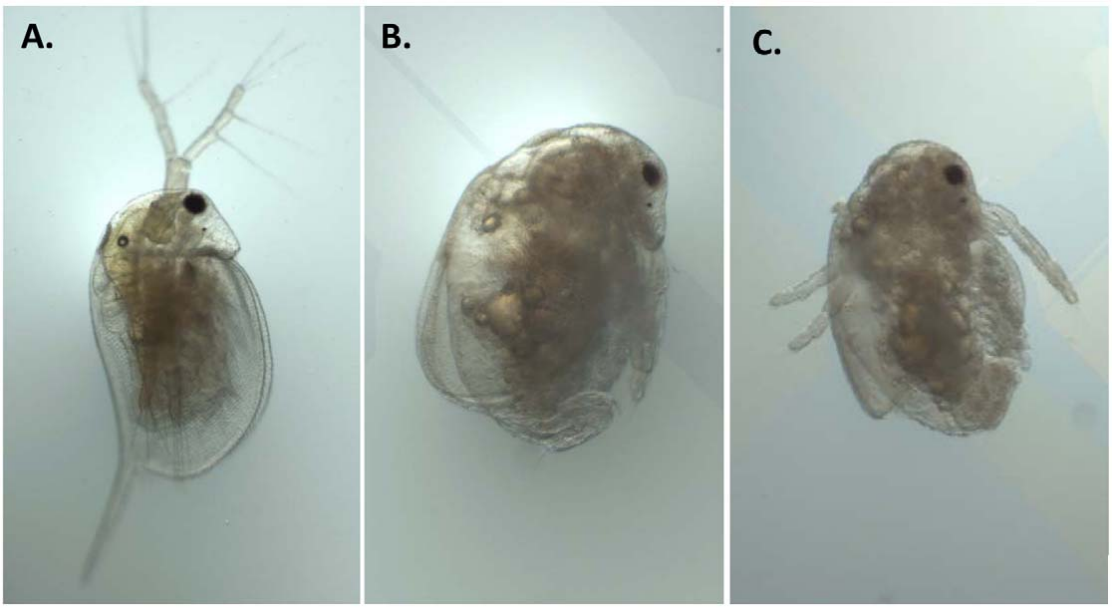
Developmental abnormalities among neonatal daphnids resulting from maternal exposure to NaNO_2_. Images presented are (**A**) normal control neonate, (**B**) and (**C**) neonates derived from maternal exposure to 1.0 and 2.0 mg N/L NaNO_2_, respectively.

Both NO_2_^−^ and NO_3_^−^ elicited reproductive and developmental toxicity that qualitatively mimicked that of sodium nitroprusside. NO_2_^−^ decreased the reproductive capacity of the daphnids (Fig. 4B) and increased the incidence of developmental abnormalities (Fig. 5B) with approximate threshold effect concentrations of 0.64 and 0.33 mg N/L, respectively. Developmental abnormalities were the same as described with sodium nitroprusside (Fig. 6). NO_3_^−^ was a significantly less potent in eliciting reproductive and developmental toxicity. NO_3_^−^ reduced the number of offspring produced with an approximate threshold effect concentration of 123 mg N/L (Fig. 4C) and increased the incidence of developmental abnormalities with a threshold effect concentration of approximately140 mg N/L (Fig. 5C). Consistency in the effects observed between sodium nitroprusside and NO_x_ supports the premise that reproductive and developmental toxicity of NO_x_ are due to the generation of nitric oxide. As noted above, lower potency of NO_3_^−^ as compared to NO_2_^−^ is likely due to its reduced uptake by the organisms and requirement for more extensive metabolism to generate nitric oxide.

### Ecdysteroid measurements

Various sodium nitroprusside exposure scenarios were executed with embryonic, juvenile, or adult daphnids to determine if developmental and reproductive toxicity of nitric oxide was due to the lowering of ecdysteroid levels (Table 1). Ecdysteroid levels detected by RIA were not significantly different between untreated (control) and sodium nitroprusside-exposed daphnids following most exposure scenarios tested. NO_x_ effects on ecdysteroid levels were not evaluated since these experiments provided no evidence that nitric oxide reduces ecdysteroids levels in daphnids.

**Table 1:** Ecdysteroid levels in daphnids exposed to 1.0 mg N/L sodium nitroprusside (SNP).

Age at start of exposure	Life stage analyzed	Exposure duration (days)	Exposure group	Individuals per group	Ecdysteroid (pg/individual)
isolated embryos	F_1_ embryos (exposed *ex vivo*)	3	Control	39–55	1.6±0.3
			SNP	44–56	1.5±0.2
8 days	F_1_ embryos (isolated from exposed F_0_ daphnids)	8	Control	65–77	1.9±2.3
			SNP	53–78	1.1±1.0
3–4 days	F_0_ daphnids	3	Control	10	106.6±4.7
			SNP	10	131.4±14.7
<24 hours	F_0_ daphnids	6	Control	10	125.3±14.4
			SNP	10	129.4±10.9
<24 hours	F_0_ daphnids	9	Control	7–10	138.1±33.5
			SNP	7–10	115.1±22.8

Ecdysteroid (pg/individual) values represent the mean ± SD (n = 3–5 treatment groups, with numbers of individuals per group as indicated).

## Discussion

The objectives of this study were: a) to evaluate the ability of arthropods to convert NO_x_ to nitric oxide; b) to determine whether NO_x_ elicit toxicity consistent with that of nitric oxide; and c) to determine whether nitric oxide elicits reproductive and developmental toxicity by lowering ecdysteroids titers. Results revealed that NO_x_ are indeed reduced to nitric oxide by arthropod cells and that NO_x_ elicit reproductive and developmental toxicity that is consistent with toxicity elicited by nitric oxide. However, no strong evidence was provided to indicate that nitric oxide elicits reproductive and developmental toxicity by lowering ecdysteroids titers.

Until recently, NO_3_^−^ was generally excepted to be biologically inert in animals and only converted to NO_2_^−^ within the body by commensal bacterial nitrate reductase [25]. However, enzymatic serial reduction of NO_3_^−^ to NO_2_^−^ to nitric oxide was detected in mammalian tissues under normoxic conditions by xanthine oxidoreductase [26]. Therefore, detection of nitric oxide production following NO_3_^−^ administration to arthropod cells suggests that similar reductase activity may be present. Preliminary experiments conducted in our laboratory have revealed that NO_2_^−^-dependent nitric oxide production in these cells is inhibited by potassium cyanide. This finding suggests that mitochondrial cytochrome *c* reductase plays a role in NO_2_^−^ reduction, as reported previously in other cell types [12], [27]. Additional mechanistic evaluation is required however to identify the source of nitrate reductase activity in arthropod cells.

At sufficiently low doses, exogenous NO_x_ has been proposed to have beneficial effects in mammals as a bactericide [28], in enhancing blood flow (vasodilation) [29], and in preventing ischemia-reperfusion cellular infarction in heart tissue [30]. Accordingly, exogenous NO_x_ from dietary sources may benefit health [31]. However, aquatic organisms may be at risk from NO_x_ as nitrates and nitrites are the most ubiquitous and abundant contaminants in freshwater and coastal ecosystems [32]. Nitrogen pollution has been associated with various environmental impacts including the alteration of food webs and loss of biodiversity [33]. These consequences of nitrogen pollution have been largely attributed to eutrophication. However, nitric oxide contributes substantially to the regulation of numerous reproductive functions in both vertebrates and invertebrates [34] and the intracellular conversion of excess nitrates and nitrites to nitric oxide in aquatic environments may contribute to the adverse consequences of nitrogen pollution. The threshold concentrations followed by steep concentration:response relationships for developmental abnormalities delineate the point at which adverse effects of nitrite and nitrate clearly surpass any beneficial effect in these organisms.

NO_x_ elicited developmental toxicity that was consistent with that caused by sodium nitroprusside. These results suggest that NO_2_^−^ may be converted to nitric oxide at sufficient levels to disrupt normal development. Nitric oxide trapping and fluorescent visualization demonstrated that NO_2_^−^ is indeed converted to nitric oxide *in vivo*. The developmental abnormalities elicited by NO_x_ were reminiscent of effects elicited by compounds that interfere with normal ecdysteroid signaling [35], [36], [37]. In arthropods, ecdysteroids function to regulate molting, reproduction, and embryonic development [38]. Anti-ecdysteroidal compounds may act by reducing ecdysteroids titers, competing antagonistically for the ecdysteroids receptor, or modulating the availability or activity of downstream contributors to the signaling cascade. We viewed binding of nitric oxide to the heme groups of cytochrome P450 enzymes involved in ecdysteroid synthesis as a particularly attractive mechanism of anti-ecdysteroidal activity as precedence exists for NO_x_ reducing steroidogenesis [10]. However, we found no compelling evidence that nitric oxide decreased ecdysteroid titers. Thus, other mechanisms by which nitric oxide may disrupt reproduction and development should be considered. Mechanisms of action to consider in future studies include oxidative damage elicited by the nitric oxide metabolite peroxynitrite [39], altered activity of nitric oxide-binding transcription factors [40], and altered guanylyl cyclase activity [41].

NO_2_^−^ was clearly more toxicologically significant than was NO_3_^−^, due to its reproductive and developmental toxicity at low exposure levels. NO_2_^−^ is considered to be a short-lived intermediate molecule in the aquatic environment [42]. However, it is readily taken up by aquatic organisms through the Cl^−^/HCO_3_^−^exchanger, an active uptake mechanism [43]. NO_2_^−^ is produced in the environment as the result of ammonium (NH_4_^+^) oxidation [44], or bacterial nitrate reduction in low-oxygen environments [45]. NO_2_^−^ levels in surface waters are not typically measured. Rather, NO_3_^−^ or total NO_x_ concentrations are more commonly reported. In multiple samplings along the Neuse River, North Carolina USA, NO_2_^−^ and NO_3_^−^ levels averaged 16 and 84%, respectively, of the total NO_x_ load in the river [46]. Turner et al. reported total NO_x_ levels in the Mississippi River, USA as high as 12 mg N/L with average yearly levels being about half that level [47]. Applying the NO_2_^−^:NO_3_^−^ ratio derived from the Neuse River data, average to peak levels of NO_2_^−^ in the Mississippi River are expected to be 1–2 mg N/L. Consistent with these expectations, Harris and Smith [48] reported NO_2_^−^ concentrations as high as 1.63 mg N/L in the Powder River Basin tributary of Wyoming, USA. Assuming that daphnids serve as a model for other crustaceans, this concentration of NO_2_^−^ would be likely to cause significant developmental and reproductive alterations in indigenous crustacean populations.

Toxicity due to chronic NO_3_^−^ exposure occurs at higher concentrations than levels typically detected in aquatic environments. However, this compound can undergo bacterial reduction in the environment to the much more potent NO_2_^−^. Often, aquatic systems with elevated nitrogen levels have depleted dissolved oxygen levels as a result of eutrophication [49]. Bacteria are capable of reducing NO_3_^−^ to NO_2_^−^ in low oxygen conditions [50], [51] under which NO_3_^−^ is used as an electron acceptor during respiration. Therefore, NO_3_^−^ may serve as a reservoir for NO_2_^−^ in eutrophic environments.

Numerous examples of unexplained population declines currently exist within aquatic environments [9]. Toxicological and mechanistic information obtained in this study may provide support for the hypothesis that environmental NO_x_ could play a role in those instances. Further investigation of the mechanism behind the reproductive and developmental toxicity observed in this study will strengthen our ability to explain the effects seen with environmental NO_x_ exposures. These results support the movement to improve efficiency and reduce waste/runoff associated with nitrogen use.

## Materials and Methods

### Measurement of intracellular conversion of NO_x_ to nitric oxide

*Drosophila* Schneider S2 cells were used to evaluate the cellular conversion of NO_x_ to nitric oxide. Cells were cultured in Schneider's medium +10% heat inactivated fetal bovine serum. Nitric oxide production by cells provided NO_x_ was measured using an ISO-NO Mark II meter, equipped with a Clark-type electrode, and DUO-18 data acquisition system (World Precision Instruments, Sarasota, FL). During experiments, the cell suspension was housed inside a sealed chamber (World Precision Instruments) with minimal airspace. The electrode was immersed in the cell suspension through a port in the chamber. The potential nitric oxide liberators NaNO_2_ and NaNO_3_ (Sigma-Aldrich, St. Louis, MI, USA) were injected into the chamber with a Hamilton syringe fitted through an injection port on the chamber. All experiments were conducted at 23°C. Nitric oxide levels in the chamber were continuously measured from time of injection of NO_x_ until a state of equilibrium was reached. Equilibrium occurred when the rate of nitric oxide production was equal to the rate of nitric oxide loss. Nitric oxide-generated millivolts, at equilibrium, were converted to the concentration of nitric oxide (nM) in solution using a standard curve. Standards curves were generated according the instrument manufacturer's recommendations using KNO_2_ as a substrate under acidic conditions. Negative controls consisted of injections of deionized water or NaCl (Sigma-Aldrich) at sodium concentrations comparable to those used in the experiments with NaNO_2_ and NaNO_3_.

### Whole organism experiments

The crustacean *Daphnia magna* was used in all whole organism experiments. Daphnids were derived from cultures maintained in our laboratory for more than 15 years. The original stock was acquired from the US Environmental Protection Agency, Mid-Continent Ecology Division (Duluth, MN). Daphnid media consisted of reconstituted deionized water (192 mg/L CaSO_4_•H_2_O, 192 mg/L NaHCO_3_, 120 mg/L MgSO_4_, 8.0 mg/L KCl, 1.0 µg/L selenium and 1.0 µg/L vitamin B_12_). Cultures were maintained at a density of 50 daphnids/L media. Medium was changed and the oldest adults discarded weekly. Culture daphnids were fed twice a day with 1.0 mL (4 mg dry weight) of Tetrafin® fish-food suspension (Pet International, Blacksburg, VA, USA) and 2.0 mL (1.4×10^8^ cells) of a unicellular green algae *Pseudokirchneriella subcapitata* suspension. The algae were cultured in Bold's basal medium. All daphnid cultures and experiments were housed at 20°C with a 16-hr photoperiod. These culture conditions maintained the daphnids in the parthenogenetic reproductive phase with production of all-female broods.

### Nitric acid accumulation in daphnid embryos exposed to NO_x_

Perinatal daphnids were excised from the brood chamber of a single maternal daphnid and divided among three 1.7 mL microfuge tubes containing 0.50 mL of medium. One tube contained NaNO_x_ (1.0 or 10 mg N/L), one contained diacetylaminofluorocene (DAF, 10 µM), and the third contained the combination of NaNO_x_ and DAF. DAF fluoresces green when bound by nitric oxide. Embryos were incubated from 1 to 30 hrs, then were examined microscopically (Leica MZ16F, Leica Microsystems, Deerfield, IL, USA) under fluorescence (480 nm excitation, 510 nm emission) and images were digitally captured (Simple PCI software, Hamamatsu Corporation, Sewickley, PA, USA).

### Developmental and reproductive toxicity

Developmental and reproductive toxicity of sodium nitroprusside (Sigma-Aldrich), NaNO_3_, and NaNO_2_ were evaluated. Daphnids were individually exposed to 10% serial dilutions of each material for a total of 20–30 concentrations. A control was provided for each experiment that consisted of 10 daphnids individually exposed to assay media. The compounds were delivered as an aqueous stock to the daphnid media. Daphnids were individually exposed in 50 mL beakers containing 40 mL solution. Solutions were changed every other day. The concentration ranges of the materials were based upon preliminary experiments and concentrations of NO_3_^−^ and NO_2_^−^ spanned environmentally-relevant levels. Exposures began with daphnids <24 hours old and continued through 4 brood cycles (about 21 days). Each daphnid was fed 100 µL algae suspension and 50 µL of fish-food suspension daily until they reached 7 days old, after which they were fed 200 µL algae suspension and 100 µL fish-food suspension, daily. Daphnids were evaluated daily for survival and release of offspring. Following the release of each brood, the number of neonates in the brood was determined and individuals were evaluated under a light microscope (Leica) for the presence of gross developmental abnormalities.

### Ecdysteroid measurements

Daphnids of various life stages were exposed to sodium nitroprusside (1.0 mg N/L) as described above. For daphnid *ex vivo* embryo exposures, developmental stage 1 embryos were isolated from untreated daphnids as described previously [52]. Entire broods isolated from 2 maternal organisms were randomly assigned to wells of a 12-well tissue culture plate and treated in media containing 1.0 mg N/L sodium nitroprusside, dissolved directly into the media, or media only (controls) in 2 mL treatment solution/well. Exposure solutions were renewed every 24 hours with fresh solution. Embryos were incubated at 20°C under 16 hour photoperiod and were exposed for 72 hours. To measure ecdysteroid concentrations in embryos following maternal exposure, embryos were removed from the brood chamber of three maternal organisms per treatment and processed for analysis in groups by treatment. Daphnid ecdysteroid levels were measured by radioimmunoassay with a standard curve of 20-hydroxyecdysone as described previously [36].

### Statistical Analysis

Data generated from the reproductive and developmental toxicity assessments were analyzed using Origin 7.5 software and regression lines fitted using the sigmoidal fit function. The threshold concentration for each compound was derived from the point at which the regression line that defined the concentration-response relationship crossed within one standard deviation for the mean control value. Significant difference in ecdysteroid levels between a single treatment group and respective control was evaluated by Student's *t*-test using JMP software (SAS Institute, Cary, NC).
